# Plastic Proteins and Monkey Blocks: How Lentiviruses Evolved to Replicate in the Presence of Primate Restriction Factors

**DOI:** 10.1371/journal.ppat.1004017

**Published:** 2014-04-17

**Authors:** Kevin R. McCarthy, Welkin E. Johnson

**Affiliations:** 1 Harvard Program in Virology Harvard Medical School, Boston, Massachusetts, United States of America; 2 Boston College, Biology Department, Chestnut Hill, Massachusetts, United States of America; University of Florida, United States of America

## Introduction

Restriction factors are cellular factors that block virus infection, either by direct interaction with viral factors or by rendering the cellular environment incompatible with viral replication. Well-characterized restriction factors include SAMHD1 [1], [2], BST-2/tetherin [3], [4], APOBEC3G [5], and TRIM5α [6]. In general, viruses evolve resistance to the restriction factors of their natural hosts but may still be sensitive to homologs of the same restriction factors from other organisms. Thus, restriction factors are potentially major determinants of virus host range in nature. Much of the evidence favoring this hypothesis comes from comparative studies of the primate lentiviruses, including HIV-1, HIV-2, and the simian immunodeficiency viruses (SIV) of African primates. While cell-culture studies have deduced molecular details of restriction, comparative evolutionary analyses are helping to reveal the biological impact of restriction in nature.

## How Have Primate Lentiviruses Helped Us Better Understand Restriction Factors?

**Figure 1:**
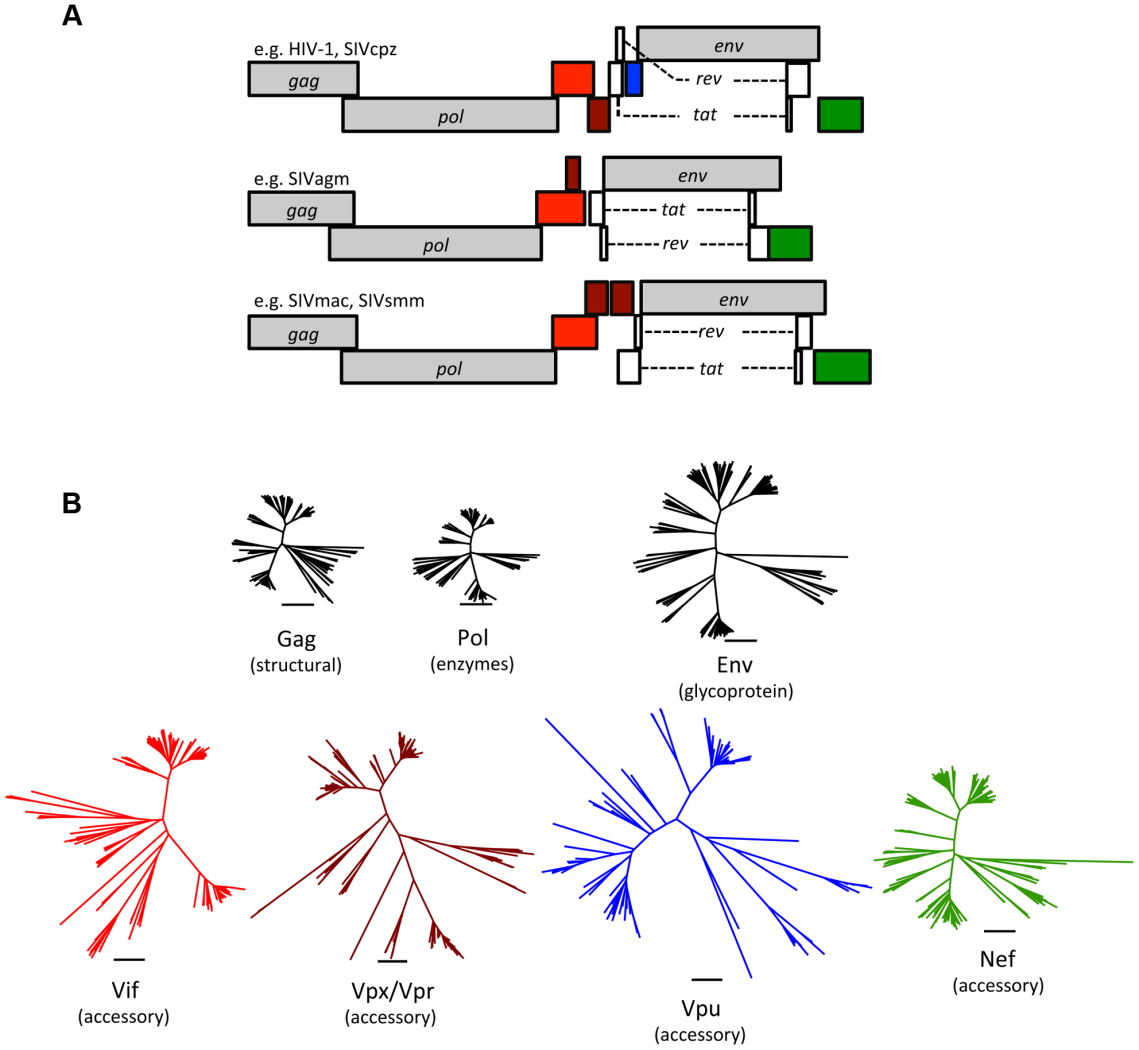
Accessory proteins are the most diverse of the primate lentivirus proteins. (A) Genomes of primate lentiviruses. Schematic representations of the three major types of genome organization found among primate lentiviruses. Genes encoding structural proteins (*gag*, *pol*, and *env*) are shown in gray. The regulatory genes, *tat* and *rev*, are in white. Accessory genes are color-coded to match the phylogenetic trees in the lower panel. (B) A comparison of genetic diversity among primate lentivirus proteins. Note that the accessory proteins are much more diverse than the structural proteins. Neighbor-Joining trees were generated using sequence alignments of primate lentiviruses available from Los Alamos National Labs (http://www.hiv.lanl.gov/). The Vpx and Vpr proteins are paralogs that arose by duplication during evolution of the primate lentiviruses and were therefore combined into a single tree.

Since the discoveries of HIV-1 and HIV-2 in humans and SIVmac in captive macaques, over 40 primate lentiviruses have been identified, all in African primates [7]. Phylogenetic comparisons revealed that HIV-1 groups M, N, O, and P arose by transmission of ape viruses (SIVcpz and SIVgor) to humans, while lentiviruses from sooty mangabeys (SIVsmm) also jumped to humans, giving rise to multiple HIV-2 groups [7]. Accidental transmission of SIVsmm occurred in colonies of captive macaques in the United States, emerging as SIVmac [7], [8]. In addition to these documented events, phylogenetic analyses suggest that the natural history of primate lentiviruses is rife with cross-species transmission events [7]. These complex retroviruses encode multiple accessory proteins that interfere with restriction, including Vif, Vpx, Vpr, Vpu, and Nef (Figure 1; Table 1). Thus, comparative studies of the primate lentiviruses and their hosts can shed light on the evolutionary and biological significance of restriction.

**Table 1:** Primate lentiviruses have an expanded repertoire of accessory genes.

Virus	Type	Genus	Structural Genes	Regulatory Genes	Accessory Genes	Counteracts
MLV	Simple	*Gammaretrovirus*	*gag*	None	None	
		(rodent)	*pol*			
			*env*			
HTLV-1	Complex	*Deltaretrovirus*	*gag*	*tax*	*hbx*	
		(primate)	*pol*	*rex*	*orfi-p12*	
			*env*		*orfii-p13*	
MVV	Complex	*Lentivirus*	*gag*	*tat*	*vif*	*APOBEC3*
		(ovine)	*pol*	*rev*		
			*env*			
HIV-1	Complex	*Lentivirus*	*gag*	*tat*	*vif*	*APOBEC3*
		(primate)	*pol*	*rev*	*vpr*	
			*env*		*vpu*	*BST2*
					*nef*	
SIVagm	Complex	*Lentivirus*	*gag*	*tat*	*vif*	*APOBEC3*
		(primate)	*pol*	*rev*	*vpr*	*SAMHD1*
			*env*		*nef*	*BST2*
SIVmac	Complex	*Lentivirus*	*gag*	*tat*	*vif*	*APOBEC3*
		(primate)	*pol*	*rev*	*vpr*	
			*env*		*vpx*	*SAMHD1*
					*nef*	*BST2*

Abbreviatons: MLV, murine leukemia virus; HTLV-1, human T cell lymphotropic virus type 1; and MVV, maedi-visna virus.

## How Does a Virus Accessorize for (Evolutionary) Success?

All retroviruses share a core set of genes: *gag*, *pro*, *pol*, and *env* (Table 1). *Gag* encodes virion structural proteins; *pol*, the viral enzymes; and *env*, the viral glycoproteins. Complex retroviruses, including lentiviruses, encode a variable number of additional proteins that serve a variety of modulatory functions. Null mutations in these accessory genes often result in attenuation of replication in vivo, even when there is little or no effect on virus replication in cell culture [9]. The accessory genes are clustered along with *env* in the 3′ half of the viral genome, separate from *gag*, *pro*, and *pol* (Figure 1). This segregation is not unique to complex retroviruses, and analogous arrangements are found in many other viruses. It is tempting to speculate that physical separation of genes encoding conserved functions (structural proteins and enzymes) from variable functions (accessory proteins and surface glycoproteins) allows greater adaptive flexibility.

The accessory proteins are the most divergent lentivirus proteins (only the Env protein displays similar levels of diversity) (Figure 1). The complement of accessory genes is not identical for all primate lentiviruses; for example, several do not have a *vpx* gene (e.g., HIV-1 and SIVcpz), and many do not have *vpu* (e.g., SIVsmm and SIVmac) (Figure 1). If the comparison is expanded to include the “nonprimate” lentiviruses (e.g., lentiviruses found in cats, cattle, horses, and small ruminants), a distinct set of accessory genes is found, with only *vif* being common to both primate and nonprimate lentiviruses (Table 1). Thus, lentivirus accessory genes vary in primary sequence and overall composition. Such evolutionary plasticity is consistent with the notion that accessory genes help determine species tropism and may play a role in interspecies transmission and the emergence of lentiviruses.

## Are No Two Restriction Factors Alike?

Comparative studies of HIV-1 and the other primate lentiviruses have revealed several host proteins that restrict viral replication. Four of these, SAMHD1, BST-2/tetherin, APOBEC3G, and TRIM5α, have been particularly well characterized and serve to demonstrate the diversity of restriction mechanisms.

### TRIM5

The block imposed by TRIM5α occurs after viral entry but prior to provirus integration, whence it binds capsid cores in the cytoplasm and promotes premature uncoating and degradation [6], [10], [11]. Thus, the activity of TRIM5α is akin to cracking open the retroviral “egg” before the reverse transcriptase complex is ready to hatch. How TRIM5α recognizes divergent retroviruses is unknown but probably involves multimerization on the surface of incoming capsids through a series of high-avidity and low-affinity interactions [10]–[12]. Of the four examples, it is noteworthy that no viral antagonists of TRIM5α have been reported. Instead, resistance (or sensitivity) to TRIM5α is determined directly by variation in the viral capsid structure [6], [13], [14].

### SAMHD1

SAMHD1, a deoxynucleoside triphosphate triphosphohydrolase, is targeted by lentiviral Vpx proteins [1], [2]. SAMHD1 depletes deoxyribonucleotide triphosphate (dNTP) pools within terminally differentiated target cells, such as the macrophage, thereby starving the reverse transcription complex of dNTPs [15]–[17]. Curiously, HIV-1 (and some other lentiviruses) do not encode Vpx or its functional equivalent, even though Vpx provided in trans dramatically enhances HIV-1 replication in the macrophage [15].

### BST2

BST2 (also known as tetherin, CD31, or HM1.24) “traps” newly budded virions as they emerge from the surfaces of infected cells, even after complete scission of the virion and cellular membranes [3], [4], [11], [15]. These tethered virions are then removed from the cell surface by endocytosis and degraded inside the cell.

### APOBEC3G

APOBEC3G is a cytosine deaminase that targets single-stranded DNA [5], [11], [15]. APOBEC3G expressed in the infected cell is incorporated into newly assembling retrovirus virions [5], [11], [15]. Reverse transcription in the next target cell produces a minus-strand DNA intermediate, which is attacked by APOBEC3G. Deamination of cytosines to uracils in the viral minus-strand DNA produces C-to-U mutations, resulting in a lethal dose of G-to-A substitutions in the coding strand [11], [15]

## What Happens When a Virus Accessory Protein Meets a Host Restriction Factor?

SAMHD1, BST-2, and APOBEC3G are evolutionarily and mechanistically distinct from one another but share a common feature: the means by which they inhibit viruses are relatively nonspecific. For example, SAMHD1 inhibits viral replication indirectly by limiting the availability of precursors of DNA synthesis [1], [2], [15]–[17], potentially affecting any virus for which DNA synthesis is essential [18]. Likewise, BST-2 can, in theory, “capture” any membrane-enveloped structure (e.g., a virion) as it buds from the cell surface [11], [15]. APOBEC3G acts on single-stranded DNA produced during reverse transcription, but there is no evidence that the enzyme has a selective preference for viral DNA over other single-stranded DNAs. In other words, none of these factors targets a specific viral protein, and consequently, viral resistance does not result from escape mutations in a binding site or epitope. Instead, all three factors are targeted by viral accessory proteins, and interactions with these viral antagonists are primary determinants of viral sensitivity to restriction.

The Vif and Vpx proteins use similar mechanisms to overcome restriction by APOBEC3 and SAMHD1, respectively. In both cases, the viral protein couples its target to ubiquitin-ligase complexes, resulting in proteasome-mediated degradation of the restriction factor [11], [15]. Vpu also engages cellular ubiquitin ligase complexes, which may contribute, in part, to removal or sequestration of BST-2/tetherin from the cell surface. Vpr is a paralog of Vpx, and like Vpx, it interacts with the cellular ubitquitin-ligase machinery [1], [2], [19]. The cellular target of HIV-1 Vpr remains to be discovered, although in some SIV lineages Vpr has anti-SAMHD1 activity [20].

Lentiviral Nef proteins modulate cell-surface expression of many cellular proteins, and for some SIV strains, Nef is the primary antagonist of BST-2 [21], [22]. Interestingly, when SIVsmm jumped to humans and became HIV-2, the Nef protein could not interact with human BST-2 [7], [21], [22]. Consequently, the Env protein of HIV-2 evolved the capacity to counteract human BST-2 [23]. Similarly, SIVcpz Nef cannot engage human BST-2, and emergence of HIV-1 involved adaptation of Vpu to take on this function [7], [21], [22].

## What Can Molecular Evolution Tell Us about the Significance of Restriction Factors?

SIVmac arose by unintentional transmission of SIVsmm from African mangabeys to Asian macaques in captivity. Because of similarities to HIV infection and AIDS, SIV infection of macaques is a major animal model for AIDS research. SIV strains with accessory genes inactivated (individually and in different combinations) are significantly attenuated in macaques, the first hint that the accessory functions are important in vivo [9]. One group retrospectively analyzed macaques that had been vaccinated with an SIV strain lacking *nef* [24]. The virus in these animals acquired adaptive changes in *env*, giving the viral glycoprotein antitetherin activity and making up for the loss of Nef—a case of neofunctionalization belying the in vivo significance of tetherin-mediated restriction. Historical emergence of SIVmac in macaques also required adaptations in capsid, rendering it resistant to macaque homologs of TRIM5α [13], and in Vif, conferring the ability to target macaque alleles of APOBEC3G for degradation [25]. Similar adaptations occur in macaques experimentally infected with strains of SIVsmm [13], [26]

If viruses have had a major impact on host evolution, it is reasonable to expect that host genes encoding restriction factors will bear signatures of selection by viral pathogens. Indeed, this is the case, and there is very strong evidence that ancient selective events occurred during primate evolution involving all four factors [19], [27], [28]. Most strikingly, individual residues that show the most variability between species are often those that interact physically with viral targets or viral antagonists. The assumption that similar interactions were responsible for selective events in the past, coupled with phylogenetic and molecular clock analyses, has pushed the estimated age of SIVs from less than 1 million years to at least 8–15 million years [14], [28], [29]

## Summary

Taken together, the existence of viral accessory proteins dedicated to thwarting restriction, evidence that restriction factors evolve under positive selection, and the in vivo impact of restriction in SIV and AIDS models strengthen the hypothesis that restriction factors are major determinants of host range in nature, acting as selective barriers to cross-species transmission of viral pathogens. Novel mechanisms of restriction continue to be discovered. For example, Schlafen-11 exploits differences in human and viral codon usage to restrict HIV-1 [30], and MX2/MXB is a capsid-sensing component of the interferon-induced block to HIV-1 infection [31]–[33]. As more restriction factors are identified and their mechanisms deduced, it will be interesting to ask whether the interplay between restriction factors and viruses is a dominant theme in cross-species transmission, adaptation, and emergence of viruses.
